# DEL-1: a promising treatment for AMD-associated ER stress in retinal pigment epithelial cells

**DOI:** 10.1186/s12967-024-04858-9

**Published:** 2024-01-09

**Authors:** ChangHyuk Kwon, Wonjun Cho, Sung Woo Choi, Heeseung Oh, A. M. Abd El-Aty, Ibrahim Gecili, Ji Hoon Jeong, Tae Woo Jung

**Affiliations:** 1 GenesisEgo, Seoul, Republic of Korea; 2 Department of Pharmacology, College of Medicine, Chung-Ang University, 221, Heuksuk-dong, Dongjak-gu, Seoul, 156-756 Republic of Korea; 3 Department of Pharmacology, Faculty of Veterinary Medicine, Cairo University, Giza, 12211 Egypt; 4 Department of Medical Pharmacology, Medical Faculty, Ataturk University, 25240 Erzurum, Türkiye; 5 Department of Global Innovative Drugs, Graduate School of Chung-Ang University, Seoul, Republic of Korea

**Keywords:** DEL-1, Retinal pigment epithelial cells, AMD, ER stress, Autophagy, Apoptosis

## Abstract

**Background:**

Age-related macular degeneration (AMD) is an irreversible eye disease that can cause blurred vision. Regular exercise has been suggested as a therapeutic strategy for treating AMD, but how exercise improves AMD is not yet understood. This study investigated the protective effects of developmental endothelial locus-1 (DEL-1), a myokine upregulated during exercise, on endoplasmic reticulum (ER) stress-induced injury in retinal pigment epithelial cells.

**Methods:**

We evaluated the levels of AMPK phosphorylation, autophagy markers, and ER stress markers in DEL-1-treated human retinal pigment epithelial cells (hRPE) using Western blotting. We also performed cell viability, caspase 3 activity assays, and autophagosome staining.

**Results:**

Our findings showed that treatment with recombinant DEL-1 dose-dependently reduced the impairment of cell viability and caspase 3 activity in tunicamycin-treated hRPE cells. DEL-1 treatment also alleviated tunicamycin-induced ER stress markers and VEGF expression. Moreover, AMPK phosphorylation and autophagy markers were increased in hRPE cells in the presence of DEL-1. However, the effects of DEL-1 on ER stress, VEGF expression, and apoptosis in tunicamycin-treated hRPE cells were reduced by AMPK siRNA or 3-methyladenine (3-MA), an autophagy inhibitor.

**Conclusions:**

Our study suggests that DEL-1, a myokine, may have potential as a treatment strategy for AMD by attenuating ER stress-induced injury in retinal pigment epithelial cells.

## Background

As life expectancy rises globally, the incidence of blindness caused by macular degeneration is increasing [1]. While oxidative stress has long been considered a major factor in macular degeneration, the role of endoplasmic reticulum (ER) stress has also been discussed in recent years [2]. There are two types of macular degeneration, atrophic and exudative, depending on the presence or absence of choroidal neovascularization. The disease is classified into early and late stages based on the pathological period, with early age-related macular degeneration (AMD) characterized by the formation of subretinal vitreous membrane drusen in the macular region, which gradually increases in size over time [1]. The accumulation of toxic metabolites further worsens the condition, eventually leading to late-stage AMD, known as atrophic and exudative AMD. Atrophic AMD accounts for over 90% of AMD cases, with exudative AMD comprising the remaining 10% [3, 4]. Vascular endothelial growth factor (VEGF) directly promotes choroidal neovascularization, leading to bleeding and disruption, ultimately worsening vision and resulting in exudative AMD. Therefore, targeting VEGF is recognized as a therapeutic strategy for treating AMD. Therefore, anti-VEGF monoclonal antibodies have been developed to treat exudative AMD [4]. However, this approach is ineffective for AMD with choroidal neovascularization, and repeated intravitreal injections can induce a pathological condition similar to atrophic, highlighting the need for more effective treatments with fewer side effects for both atrophic and exudative AMD.

The endoplasmic reticulum (ER) is crucial in protein and lipid synthesis and serves as a calcium reservoir for intracellular calcium homeostasis [5]. However, various physical and pathological conditions, such as nutritional starvation, excessive nutrition, viral infection, and protein mutations, can accumulate misfolded or unfolded proteins in the ER lumen. Over time, these misfolded proteins can aggregate and become insoluble, causing cellular toxicity. This condition is known as ER stress, which occurs when the folding capacity of the ER is overwhelmed [6]. The pathogenesis of AMD is not fully understood, but aging [7] can contribute to prolonged ER stress [8, 9], which has been shown in several studies to be associated with the progression of AMD. Fernandez et al. demonstrated that ER stress increases the expression of VEGF, which stimulates neovascularization in the retinal pigment epithelium (RPE) in AMD [10]. Jing et al. reported elevated ER stress-mediated retinal apoptosis and cell death in AMD and diabetic retinopathy [2]. Therefore, appropriate modulation of ER stress may halt or slow the development of AMD.

Developmental endothelial locus-1 (DEL-1) is a glycoprotein that is secreted by vascular endothelial cells during embryological vascular development [11] and has recently been identified as a myokine that is upregulated during exercise [12]. DEL-1 has been shown to possess anti-inflammatory properties [12] and can alleviate ER stress in various cell types [13]. Additionally, DEL-1 has been found to attenuate lipid-induced insulin resistance through AMPK signaling [12] and hepatic steatosis via the SIRT1 pathway [13]. Based on these findings, we hypothesized that DEL-1 might benefit ER stress-mediated injury in human retinal pigment epithelium (hRPE), a cell type implicated in age-related macular degeneration (AMD). In this study, we investigated the effects of DEL-1 on ER stress, VEGF expression, and cell viability in hRPE cells using clinical data analysis as well as in vitro experimental models of AMD. We also explored the molecular mechanisms underlying the protective effects of DEL-1 against ER stress in hRPE cells.

## Methods

### Analysis of DEL-1 and CHOP mRNA expression in human tissues using high-throughput sequencing

The mRNA expression levels of DEL-1 in human myocytes and CHOP in human choroidal neovascular membranes were examined using publicly available datasets (GSE167186 and GSE146887, respectively) from the GEO Database. RNA samples were sequenced using Illumina next-generation sequencing (NGS) instruments, generating an average of 68 million paired-end reads per sample. The reads were trimmed, aligned to the human genome (Ensembl GRCh37.73), and sorted using STAR 2.3.1u and Samtools 0.1.18. Gene counts were calculated using HTSeq-count 0.5.4p3 with default settings for the subsequent differential expression analysis. Transcriptional profiles were analyzed for myocytes from 44 human subjects (19 young and 25 old) and choroidal neovascular membranes from 6 human subjects (3 healthy and 3 AMD).

### Cell culture and treatment conditions for ARPE-19 and C2C12 cells

#### hRPE cell culture

ARPE-19 (ATCC, Manassas, VA, USA) cells were cultured in RPMI-1640 medium (Gibco, Carlsbad, CA, USA) supplemented with 10% (*v/v*) fetal bovine serum (FBS) and 1% (*v/v*) antibiotics (100 U/mL penicillin and 100 μg/mL streptomycin) (HyClone, Logan, UT, USA).

### Primary human retinal pigment epithelial (hRPE) cell culture

Cultivation of primary hRPE cells obtained from Sciencell Research Laboratories (San Diego, CA, USA) was performed using optimized Epithelial Cell Medium (Sciencell Research Laboratories). This study utilized cells from early passages (3–4).

#### Mouse skeletal muscle cell culture

C2C12 mouse premyocytes were purchased from ATCC and cultured in Dulbecco's modified Eagle's medium (DMEM) (Invitrogen, Carlsbad, CA, USA) supplemented with 10% (*v/v*) FBS and 1% (*v/v*) antibiotics (HyClone). C2C12 premyocytes were cultured in DMEM containing 2% (*v/v*) horse serum (Invitrogen) to stimulate differentiation for 4 days. All cell cultures were maintained at 37 °C under 5% CO_2_, and cells within 2–4 passages were used for the study. Mycoplasma contamination was not detected. For treatment conditions, cells were treated with varying concentrations of tunicamycin (Sigma, St. Louis, MO, USA) ranging from 0 to 10 μg/mL and/or DEL-1 (Abcam, Cambridge, MA, USA) ranging from 0 to 1 μg/mL for 24 h.

### Assessment of cell viability using MTT assay

Cell viability was determined using the 3-[4,5-dimethylthiazol-2-yl]-2,5 diphenyl tetrazolium bromide (MTT) assay. Experimental cells were incubated with MTT solution (Sigma) for 1 h and then dissolved in dimethyl sulfoxide (DMSO) for 30 min. The optical density of the red formazan eluate from the cells was measured at 550 nm.

### Caspase 3 activity assay

Caspase 3 activity was assessed using a commercially available Caspase 3 Assay Kit (Sigma).

### Western blotting and antibodies

The protein expression analysis in cultured hRPE cells was conducted as follows: cultured hRPE cells were washed twice with PBS before being lysed in ice-cold PRO-PREP protein extraction solution (iNtRON Biotechnology) for 1 h. After incubation on ice, the cells in the lysis buffer were centrifuged at 13,000 rpm for 30 min at 4 °C, and the resulting supernatant was collected as the total protein extract. Total proteins were separated on SDS‒PAGE gels (10–12%) and then transferred to nitrocellulose membranes, which were blocked with 5% skim milk solution at room temperature for 1 h. The transferred membrane was incubated with primary antibodies against specific proteins, including anti-VEGF, anti-CHOP, anti-AMPK, anti-p62, and anti-β-actin (purchased from Santa Cruz Biotechnology); anti-phospho AMPK, anti-phospho eIF2α, and anti-eIF2α (obtained from Cell Signaling Technology); and anti-LC3 (purchased from Novus Biologicals). Incubation was carried out at 4 °C for 12 h. The membrane was washed three times with PBS and then incubated with matching secondary antibodies for 1 h at room temperature. After another round of washing with PBS, protein signaling was detected by exposing the membrane to an enhanced chemiluminescence solution (Bio-Rad). The protein bands were analyzed using ImageJ software, which is freely available.

### Autophagosome staining

Autophagosomes were labeled with monodansylcadaverine (MDC) to detect their presence. In brief, hRPE cells were stained with 0.1 mM dansylcadaverine (Sigma) for 20 min at 37 °C. Autophagosomes appeared as green dots and were counted in randomly selected hRPE cells.

### DEL-1 concentration levels in the cell culture medium of C2C12 myocytes

A commercial mouse DEL-1 ELISA kit (MyBioSource, San Diego, CA, USA) was utilized to quantify DEL-1 release from C2C12 myocytes following the manufacturer's instructions.

### Statistical analyses

All statistical analyses were performed using GraphPad Prism 7.0 software (GraphPad Software, San Diego, CA, USA). The results are presented as the means ± standard deviations (SDs) and expressed as arbitrary units, with the highest value set to 1 for relative comparison in experimental models. Each experiment was conducted in triplicate or more for statistical validity. Statistical significance was determined using one-way repeated ANOVA followed by Tukey post hoc tests.

## Results

### ER stress aggravates the expression of DEL-1 in skeletal muscle cells

**Fig. 1:**
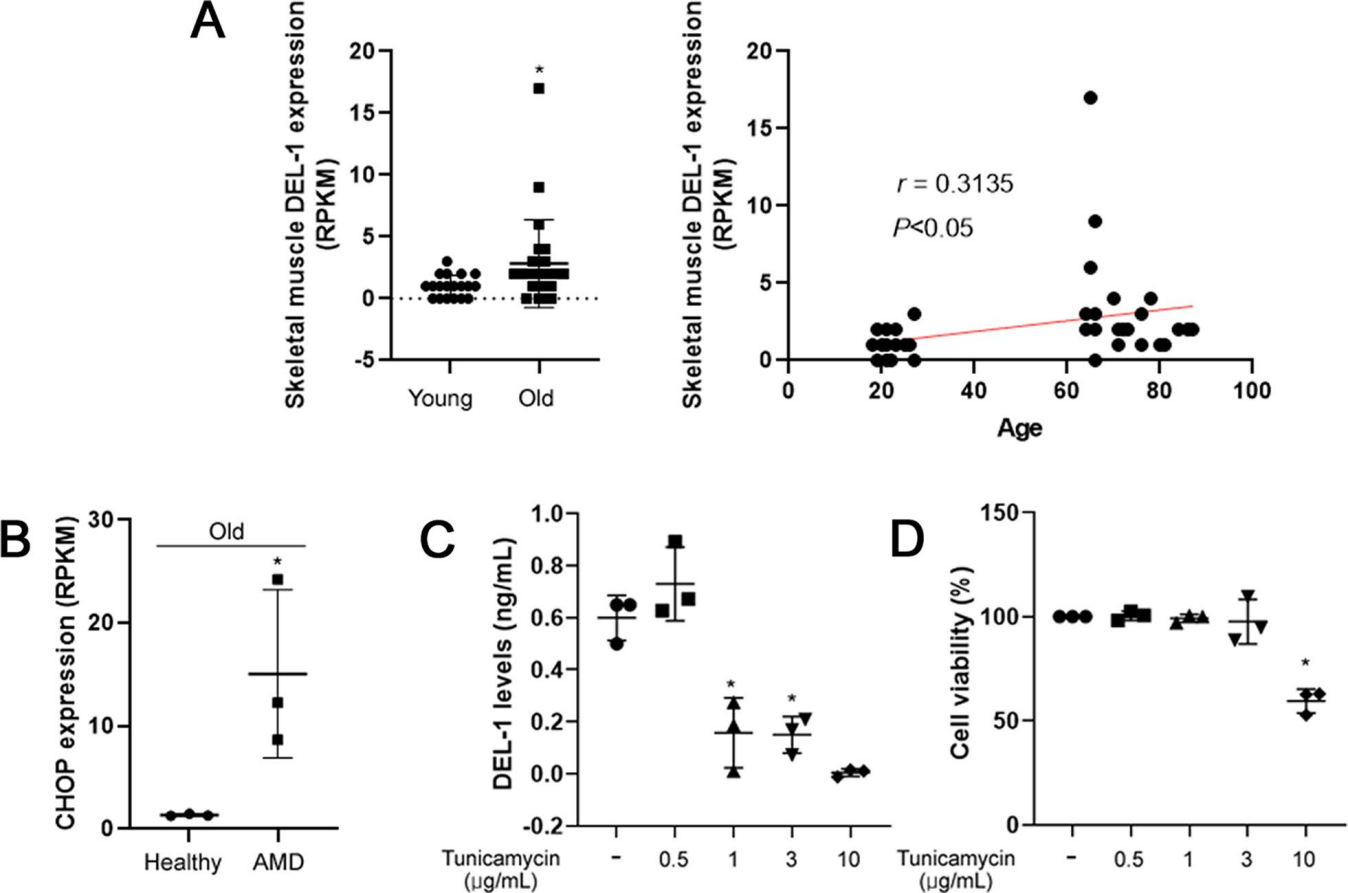
Influence of ER stress on DEL-1 expression in the skeletal muscle of AMD models. **A** RNA-seq analysis depicting DEL-1 mRNA expression in myocytes of young (n = 19) and old (n = 25) human subjects, along with the correlation between DEL-1 mRNA expression and age. **B** RNA-seq analysis illustrating CHOP mRNA expression in choroidal neovascular membranes of healthy subjects (n = 3) and patients with AMD (n = 3). **C** ELISA results showing DEL-1 levels in the culture medium of C2C12 myocytes treated with tunicamycin (0–10 μg/mL) for 24 h. **D** Cell viability assay outcomes in C2C12 myocytes treated with tunicamycin (0–10 μg/mL) for 24 h. Means ± SDs were calculated from at least three independent experiments in in vitro and clinical models. *P < 0.05 compared with young, healthy, or control

We conducted RNA-seq analysis using publicly available clinical data to investigate the potential role of the myokine DEL-1 in the pathogenesis of AMD mediated by ER stress. Our results showed a significant increase (*P* < 0.05) in DEL-1 mRNA expression levels in the skeletal muscle of old subjects compared to young subjects (Fig. 1A). Moreover, DEL-1 mRNA expression appeared to be elevated with increasing age (Fig. 1A). Additionally, retinal cells from old AMD patients exhibited higher levels of CHOP mRNA expression than those from old healthy individuals (Fig. 1B). This is consistent with previous findings by Deldicque, who demonstrated that intramuscular ER stress levels increase with age [14, 15]. To further investigate the effect of tunicamycin, an ER stress inducer, on DEL-1 expression in cultured skeletal muscle cells, we treated C2C12 myocytes with a range of tunicamycin doses (0–10 μg/mL) known to induce ER stress and apoptosis in C2C12 myocytes [15]. Interestingly, a low dose of tunicamycin (0.5 μg/mL) seemed to slightly increase DEL-1 secretion. In comparison, treatment with a higher dose of tunicamycin (1 μg/mL) downregulated basal DEL-1 release from C2C12 myocytes without affecting cell viability (Fig. 1C and D).

### DEL-1 attenuates ER stress in tunicamycin-treated retinal pigment epithelial cells

**Fig. 2:**
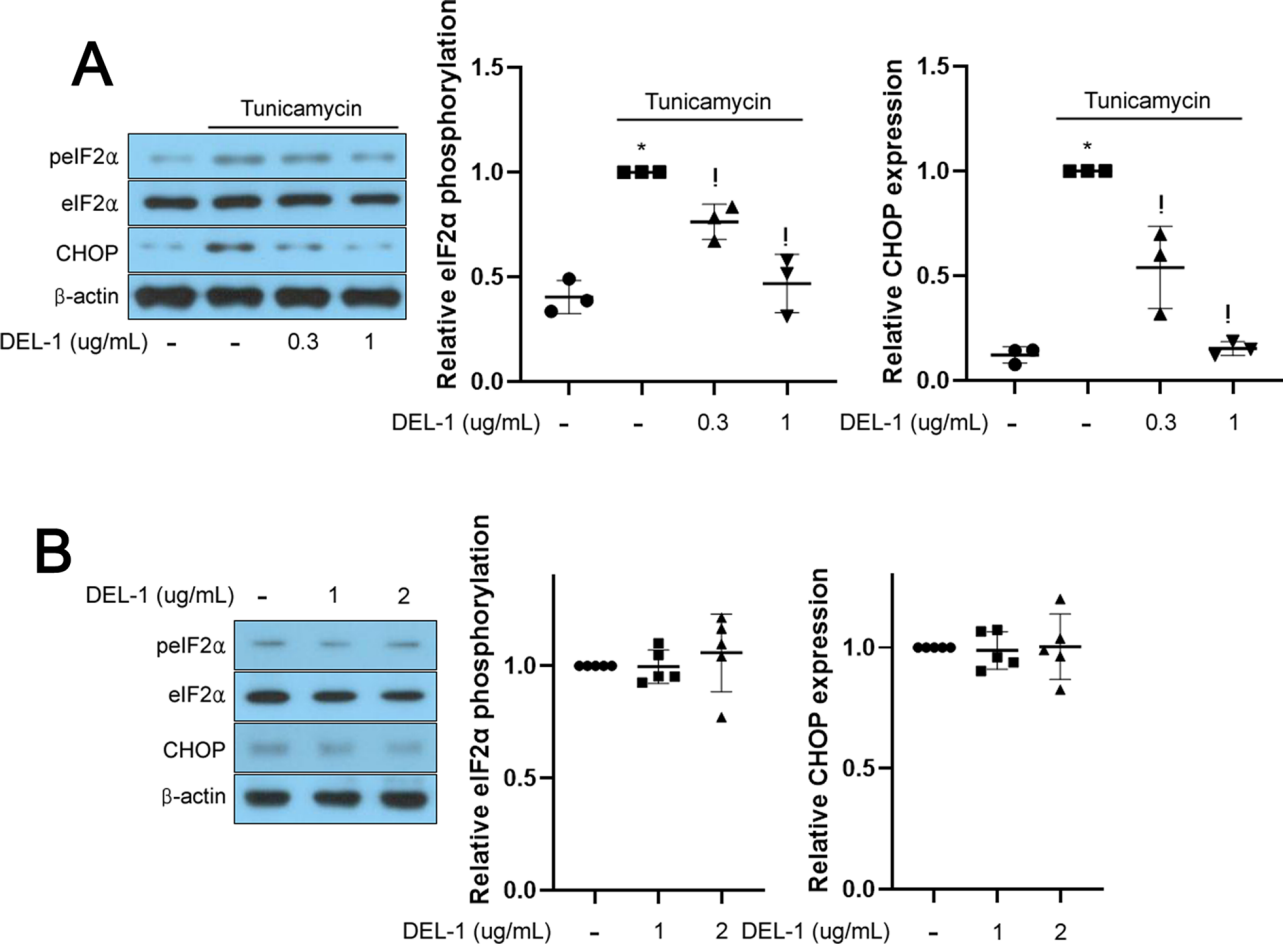
DEL-1 ameliorates ER stress in hRPE cells. **A** Western blot analysis depicting phosphorylated eIF2α and CHOP in hRPE cells treated with tunicamycin (1 μg/mL) and/or DEL-1 (0–1 μg/mL) for 24 h. **B** Western blot analysis depicting phosphorylated eIF2α and CHOP in hRPE cells treated with DEL-1 (0–2 μg/mL) for 24 h. Means ± SDs were calculated from three independent experiments in in vitro models. *P < 0.05 compared with the control. ^!^*P* < 0.05 compared with tunicamycin treatment

ER stress-induced neovascularization has been implicated in AMD, where increased apoptosis levels have been observed [2, 10]. To investigate the role of DEL-1 in ER stress-mediated AMD pathogenesis, we examined its effect on ER stress markers in hRPE cells. Treatment with DEL-1 suppressed the expression of phospho-eIF2α and CHOP, key markers of ER stress, in the presence of tunicamycin, an ER stress inducer (Fig. 2A). Furthermore, ER stress was not affected by sole treatment of DEL-1 (Fig. 2B).

### DEL-1 suppresses VEGF expression and ameliorates apoptosis in retinal pigment epithelial cells under ER stress conditions

**Fig. 3:**
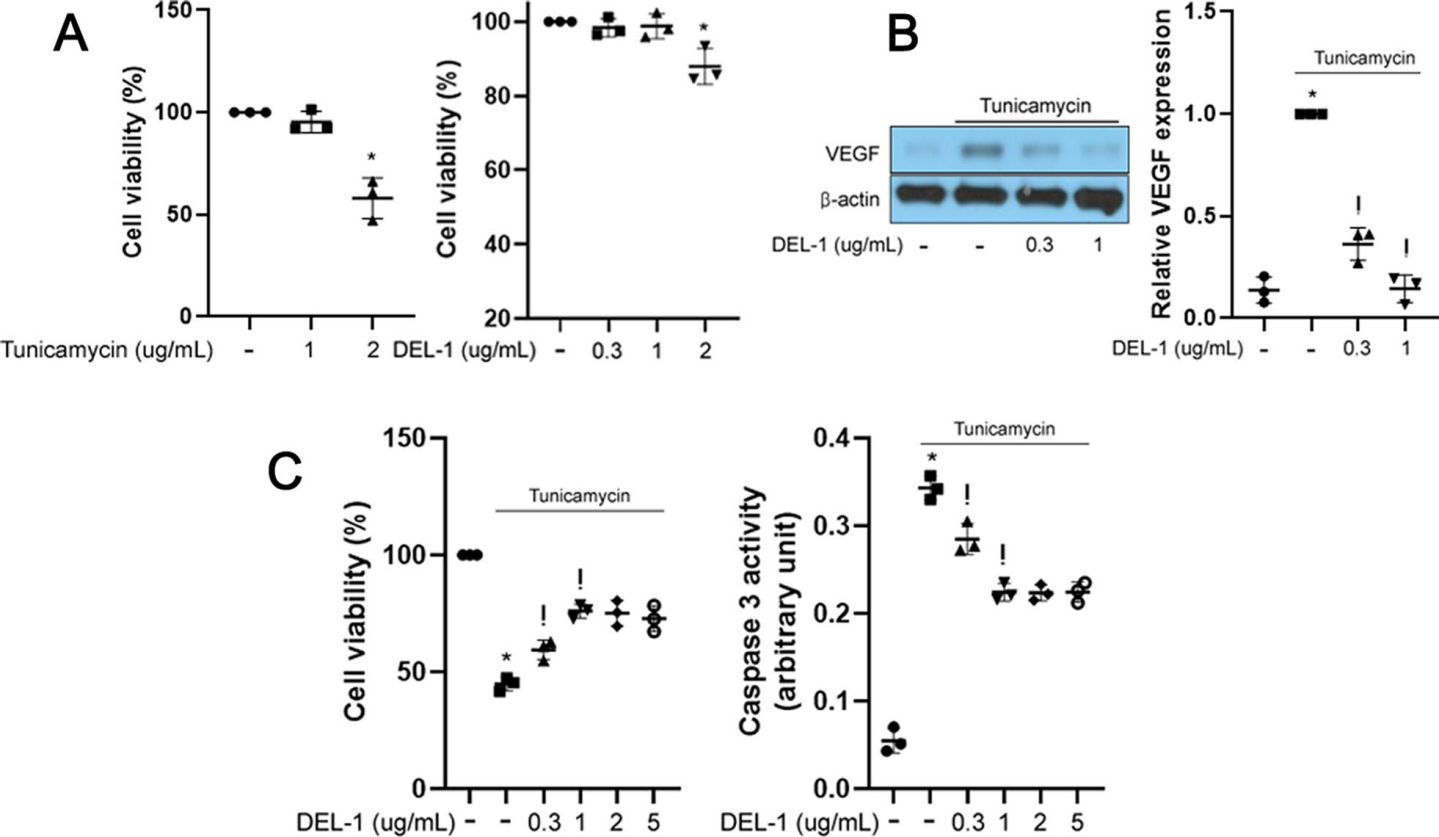
DEL-1 mitigates ER stress-induced VEGF expression and apoptosis in hRPE cells. **A** Cell viability assay in hRPE cells treated with tunicamycin (0–2 μg/mL) or DEL-1 (0–2 μg/mL) for 24 h. **B** Western blot analysis of VEGF in hRPE cells treated with tunicamycin (1 μg/mL) and/or DEL-1 (0–1 μg/mL) for 24 h. **C** Cell viability and caspase 3 activity assay in hRPE cells treated with tunicamycin (2 μg/mL) and/or DEL-1 (0–5 μg/mL) for 24 h. Means ± SDs were calculated from three independent experiments in in vitro models. *P < 0.05 compared with the control. ^!^*P* < 0.05 compared with tunicamycin (1 or 2 μg/mL) treatment

In hRPE cells, treatment with 2 μg/mL tunicamycin for 24 h resulted in a significant decrease in cell viability. In addition, the impairment of cell viability was observed in 2 μg/mL DEL-1 for 24 h (Fig. 3A). Exposure to 1 μg/mL tunicamycin, a concentration that induces ER stress without affecting cell viability, led to increased VEGF expression (Fig. 3B). In contrast, treatment with 2 μg/mL tunicamycin, a dose reducing cell viability by nearly half, resulted in decreased cell viability and induced caspase 3 activity (Fig. 3C). However, treatment with recombinant DEL-1 dose-dependently reversed these changes, as evidenced by restored VEGF expression and reduced caspase 3 activity (Fig. 3B and C). Notably, 2 and 5 μg/mL DEL-1 did not affect apoptosis in tunicamycin-treated hPRE cells (Fig. 3C).

### AMPK contributes to the effects of DEL-1 in retinal pigment epithelial cells

**Fig. 4:**
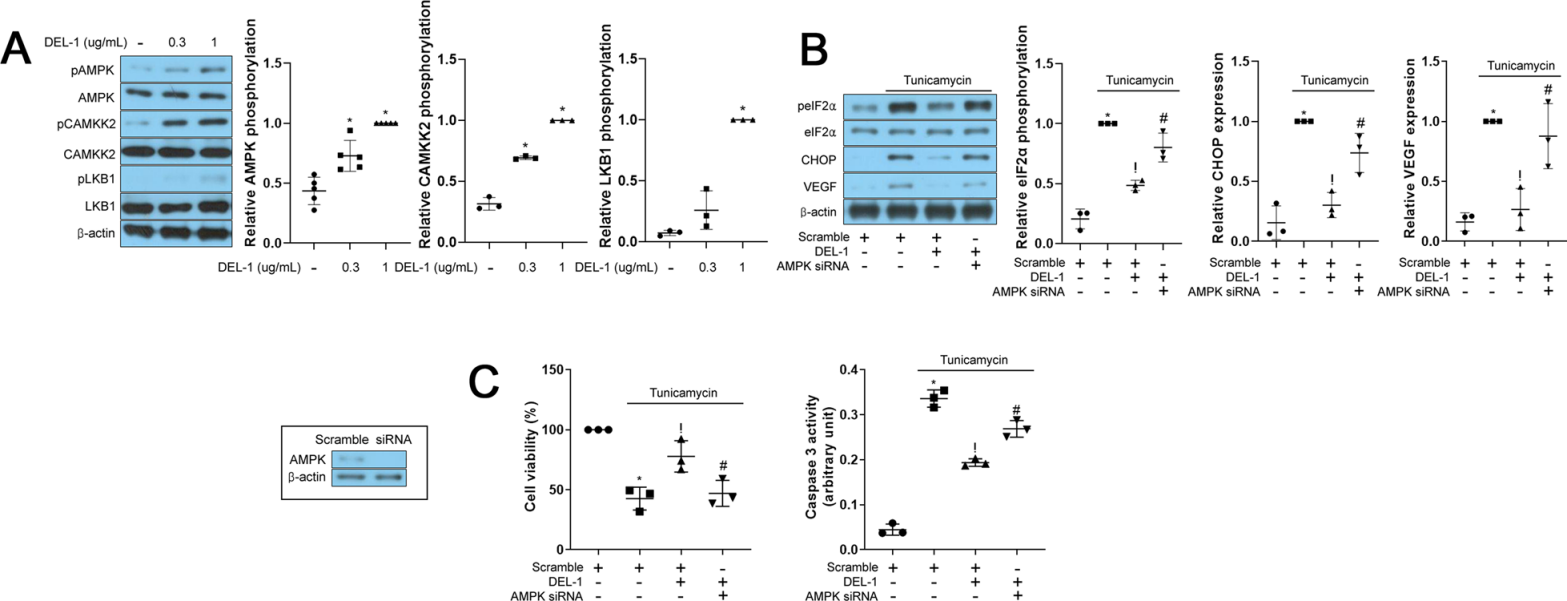
Involvement of AMPK in the effects of DEL-1 on ER stress, VEGF expression, and apoptosis in hRPE cells. **A** Western blot analysis of phosphorylated AMPK, CAMKK2 and LKB1 in hRPE cells treated with DEL-1 (0–1 μg/mL) for 24 h. **B** Western blot analysis of phosphorylated eIF2α, CHOP, and VEGF in AMPK siRNA-transfected hRPE cells treated with tunicamycin (1 μg/mL) and/or DEL-1 (1 μg/mL) for 24 h. **C** Cell viability and caspase 3 activity assay in AMPK siRNA-transfected hRPE cells treated with tunicamycin (2 μg/mL) and/or DEL-1 (1 μg/mL) for 24 h. Means ± SDs were calculated from three independent experiments in in vitro models. *P < 0.05 compared with the control. ^!^*P* < 0.05 compared with tunicamycin (1 or 2 μg/mL) treatment. ^#^*P* < 0.05 compared with tunicamycin (1 or 2 μg/mL) and DEL-1 treatment

Previous studies have reported that AMPK can alleviate ER stress in different cell types [16, 17]. In our study, we observed that treatment with DEL-1 dose-dependently enhanced AMPK phosphorylation as well as expression of phosphorylated CAMKK2 and LKB1 which are upstream molecules of AMPK in hRPE cells (Fig. 4A). Furthermore, siRNA targeting AMPK abolished the effects of DEL-1 on VEGF expression, ER stress markers, and apoptosis in hRPE cells (Fig. 4B and 4C).

### AMPK-regulated autophagy improves ER stress, thereby attenuating VEGF expression and cell injury in retinal pigment epithelial cells

**Fig. 5:**
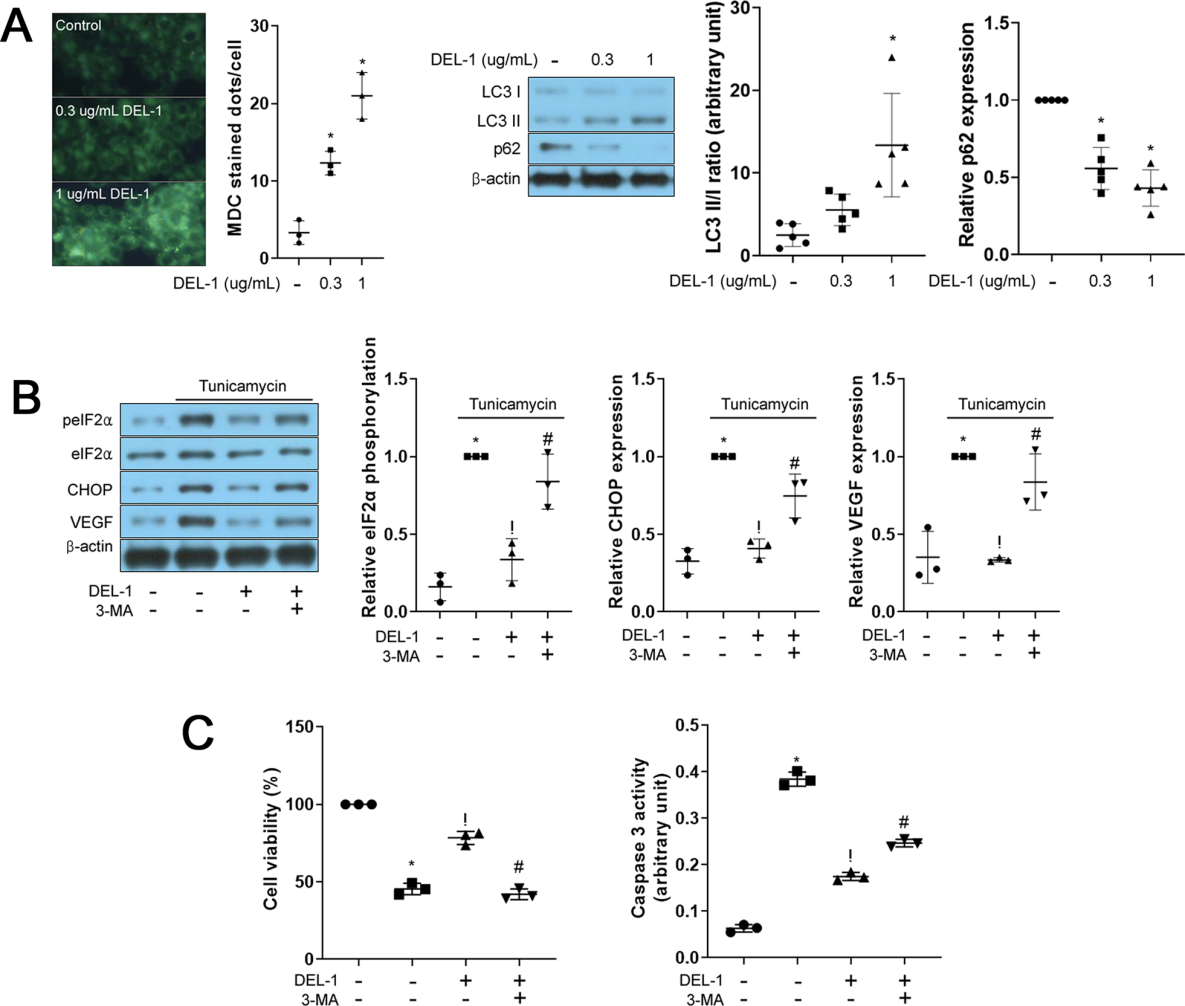
Autophagy-mediated signaling contributes to the protective effects of DEL-1 against ER stress-induced VEGF expression and injury in hRPE cells. **A** MDC staining and Western blot analysis of LC3 I/II and p62 in hRPE cells treated with DEL-1 (0–1 μg/mL) for 24 h. **B** Western blot analysis of phosphorylated eIF2α, CHOP, and VEGF in hRPE cells treated with tunicamycin (1 μg/mL), DEL-1 (1 μg/mL), and/or 3-MA (2 mM) for 24 h. **C** Cell viability and caspase 3 activity assay in hRPE cells treated with tunicamycin (1 μg/mL), DEL-1 (1 μg/mL), and/or 3-MA (2 mM) for 24 h. Means ± SDs were calculated from three independent experiments in in vitro models. *P < 0.05 compared with the control. ^!^*P* < 0.05 compared with tunicamycin (1 or 2 μg/mL) treatment. ^#^*P* < 0.05 compared with tunicamycin (1 or 2 μg/mL) and DEL-1 treatment

Autophagy has been shown to have a protective effect on ER stress in various cell types [18, 19], and it is regulated by AMPK through direct or indirect pathways [20]. Therefore, we selected autophagy as a downstream signaling target of AMPK. Treatment with DEL-1 increased autophagy markers, including autophagosome formation, LC3 conversion, and degradation of p62, in hRPE cells (Fig. 5A). Furthermore, inhibition of autophagy using 3-MA abrogated the effects of DEL-1 on VEGF expression, ER stress markers, and apoptosis in hRPE cells (Fig. 5B and 5C).

### DEL-1 alleviates ER stress and apoptosis in primary hRPE cells

**Fig. 6:**
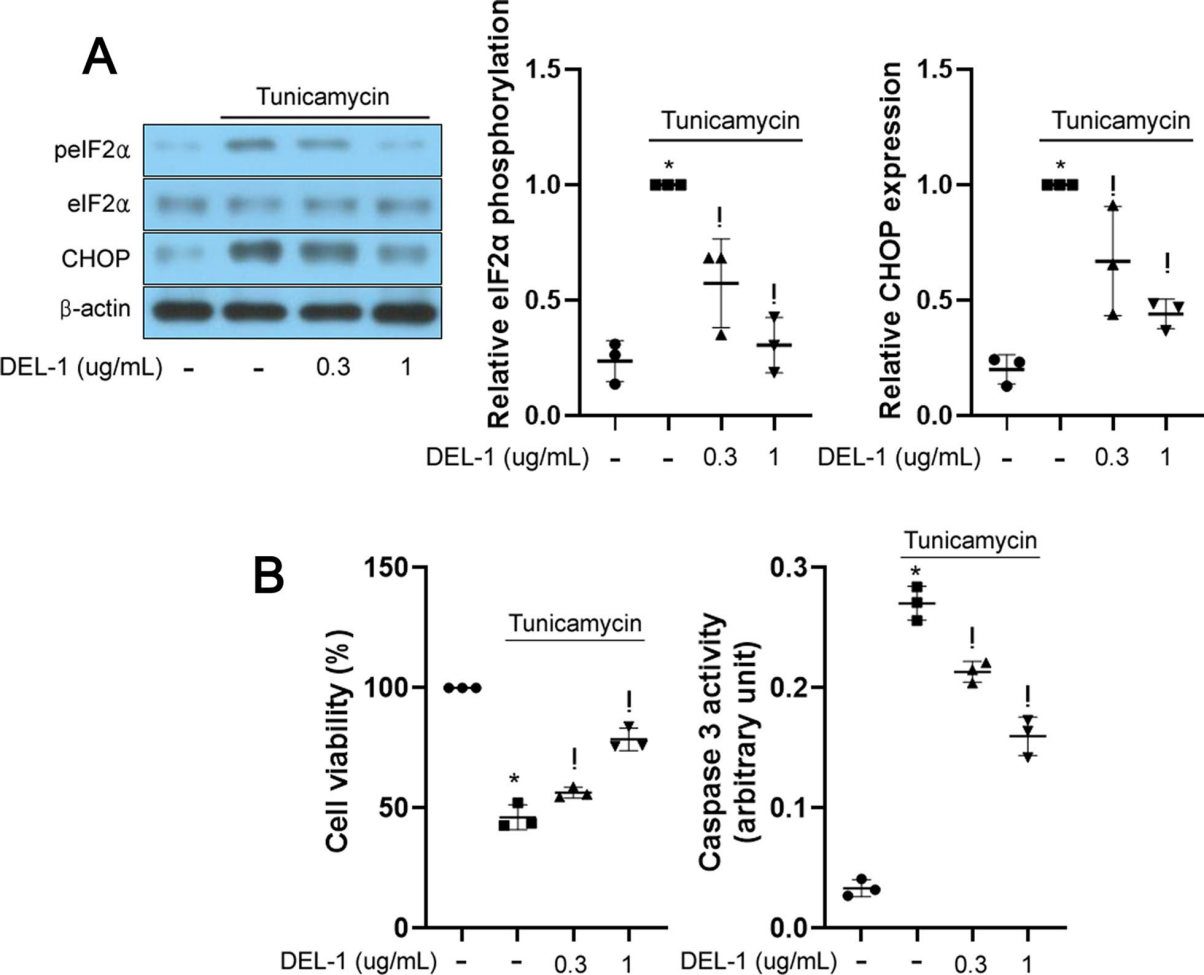
DEL-1 attenuates ER stress and apoptosis in primary hRPE cells. **A** Western blot analysis depicting phosphorylated eIF2α and CHOP in primary hRPE cells treated with tunicamycin (1 μg/mL) and/or DEL-1 (0–1 μg/mL) for 24 h. **B** Cell viability and caspase 3 activity assay in primary hRPE cells treated with tunicamycin (2 μg/mL) and/or DEL-1 (0–1 μg/mL) for 24 h. Means ± SDs were calculated from three independent experiments in in vitro models. *P < 0.05 compared with the control. ^!^*P* < 0.05 compared with tunicamycin (1 or 2 μg/mL) treatment

To replicate the in vitro findings, we extended our investigation to examine the impact of DEL-1 in primary hRPE cells. DEL-1 treatment alleviated ER stress markers and mitigated the decline in cell viability and caspase 3 activity in tunicamycin-treated primary hRPE cells, reflecting the results displayed in vitro with the ARPE-19 cell line (Fig. 6A and B).

## Discussion

AMD, a leading cause of blindness in elderly individuals, is challenging to recover from once it progresses. Therefore, developing effective treatment methods for this geriatric disease is crucial. Our study demonstrates that the myokine DEL-1 can attenuate ER stress-induced VEGF expression and apoptosis in hRPE cells, suggesting its potential as a therapeutic agent for AMD and eliminating the need for indirect methods such as exercise. Furthermore, our findings reveal the critical role of the AMPK/autophagy mechanism in the therapeutic effects of DEL-1. As DEL-1 is an endogenous protein with low side effects and toxicity, it holds promise for use as an eye drop without burdening the eyes.

In our study, we utilized RNA-seq analysis of clinical data and found that the mRNA expression levels of skeletal muscle DEL-1 increased with age. Moreover, elevated ER stress was observed in the choroidal neovascular membranes of AMD patients. These results suggest that the increased ER stress associated with aging initiates an elevation in DEL-1 levels in skeletal muscle. Nevertheless, intense ER stress ultimately inhibits DEL-1 expression in skeletal muscle cells, leading to apoptosis. Therefore, DEL-1 may function as a protective mechanism against ER stress, suggesting the possibility of mitigating AMD by improving ER stress in choroidal neovascular membranes through exogenous DEL-1. Additionally, we observed that treatment with tunicamycin at 0.5 μg/mL slightly increased DEL-1 secretion from cultured myocytes. In contrast, 1 μg/mL tunicamycin aggravated this effect, although 3 μg/mL tunicamycin did not affect the viability of muscle cells. Consistent with our RNA-seq analysis and in vitro results, treatment with DEL-1 attenuated ER stress in tunicamycin-treated hRPE cells, revealing the protective effect of DEL-1 on ER stress.

VEGF [19, 21, 22] and apoptosis [23] in retinal pigment epithelial cells are pivotal in the development of AMD. Previous studies have shown that adenovirus-mediated overexpression of VEGF leads to choroidal neovascularization in animal models [23], and hydroquinone enhances VEGF expression and promotes apoptosis in the hRPE, resulting in atrophic AMD [23]. VEGF released from the hRPE is a growth factor for retinal pigment epithelial cells and nearby vascular cells, contributing to exudative AMD [23]. Our current study found that treatment with DEL-1 attenuated tunicamycin-induced VEGF expression and apoptosis in hRPE cells in a dose-dependent manner. These findings suggest that DEL-1 may fundamentally improve both atrophic and exudative AMD by simultaneously suppressing ER stress-induced VEGF expression and apoptosis in the hRPE.

AMPK, an energy sensor that regulates ATP production, is activated in conditions that require energy, such as starvation and exercise. AMPK has been shown to have beneficial effects in insulin-resistant states such as diabetes and nonalcoholic fatty liver disease in obesity [24]. Recent studies have also suggested that AMPK has protective properties in various cell types under stress conditions, including inflammation [25], oxidative stress [26], and ER stress [27]. Kwon et al. demonstrated that DEL-1 enhances AMPK phosphorylation in adipocytes [12]. In this study, we investigated, for the first time, the effect of DEL-1 on AMPK phosphorylation in hRPE cells. Our findings showed that treatment of hRPE cells with DEL-1 dose-dependently increased AMPK phosphorylation. Moreover, siRNA-mediated suppression of AMPK abolished the effects of DEL-1 on ER stress, VEGF expression, and apoptosis in tunicamycin-treated hRPE cells. These results suggest that DEL-1 alleviates ER stress through AMPK signaling, leading to the attenuation of VEGF expression and apoptosis in hRPE cells.

Autophagy is a lysosome-mediated degradation process that removes or recycles intracellular waste, such as unfolded and misfolded proteins and dysfunctional organelles, which can cause ER stress. Therefore, autophagy is considered a defense mechanism during ER stress [28]. Furthermore, autophagy is upregulated by the AMPK-dependent mTOR or Ulk1 pathway [29]. For instance, Bachar-Wikstrom et al. showed that rapamycin-induced activation of autophagy reduced ER stress in β-cells, attenuating diabetes [19]. Resolvin D3 ameliorates ER stress through AMPK-regulated autophagy signaling, thereby attenuating skeletal muscle insulin resistance and hepatic steatosis in obese mice [16]. Barbosa reported impaired autophagic activity in the elderly, leading to ER stress [30]. This led us to hypothesize that prolonged and intense ER stress, reducing DEL-1 levels in skeletal muscle cells, could diminish autophagy. Consequently, this may contribute to AMD development by increasing VEGF expression and triggering apoptosis in retinal pigment epithelial cells. Our results showed that DEL-1 treatment enhanced autophagy markers in hRPE cells, and inhibition of autophagy with 3-MA attenuated the effects of DEL-1 on ER stress, VEGF expression, and apoptosis in tunicamycin-treated hRPE cells. These results imply that the AMPK/autophagy pathway plays a role in DEL-1's impact on ER stress-induced VEGF expression and cell damage in hRPE cells. However, considering the age-related rise in DEL-1 expression depicted in Fig. 1A and B, we propose several hypotheses. Firstly, aging may lead to increased serum DEL-1 levels but decreased local concentrations in the eye. Secondly, vascular dropout due to aging may reduce DEL-1 delivery to RPE. Lastly, aging of RPE may result in decreased DEL-1 responsiveness. To validate these hypotheses, further investigations using clinical samples are warranted.

## Conclusions

**Fig. 7:**
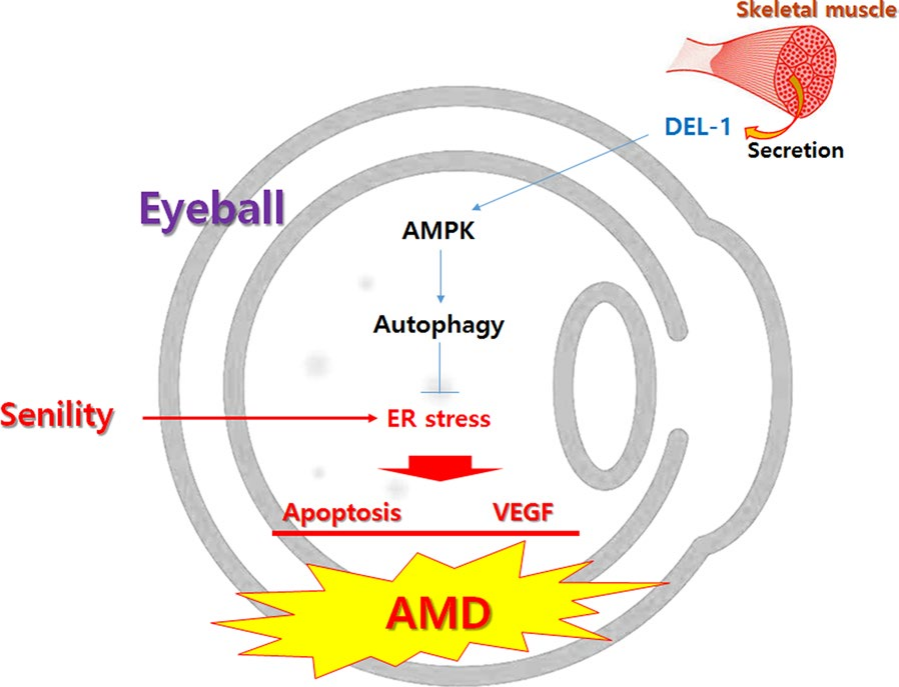
Illustration depicting the effects of the myokine DEL-1 on VEGF expression and apoptosis in hRPE cells

The findings from this in vitro study suggest that DEL-1 has the potential to attenuate ER stress-induced VEGF expression and apoptosis in hRPE cells through the AMPK/autophagy axis (Fig. 7). Further validation of these results through additional animal experiments using AMPK-deficient mice could establish DEL-1 as a promising and safe candidate for treating AMD.

## Data Availability

Data are available from the authors upon reasonable request.

## Abbreviations

AMPK: AMP-activated protein kinase
eIF2α: Eukaryotic translation initiation factor 2α
CHOP: CCAAT-enhancer-binding protein homologous protein
LC3: Microtubule-associated protein 1A/1B-light chain 3
3-MA: 3-Methyladenine
ER: Endoplasmic reticulum
AMD: Age-related macular degeneration
DEL-1: Developmental endothelial locus-1
hRPE: Human retinal pigment epithelial cells
VEGF: Vascular endothelial growth factor
